# Precisely Timed On‐Demand Droplet Ejection and Collision in Free Space for Controlled Reaction Initiation

**DOI:** 10.1002/smll.74957

**Published:** 2026-08-05

**Authors:** Hao Deng, Jacek J. Jasieniak, Tuncay Alan

**Affiliations:** ^1^ Dynamic Micro Devices Laboratory Mechanical and Aerospace Engineering Monash University Melbourne Australia; ^2^ Materials Science and Engineering Monash University Melbourne Australia

**Keywords:** droplet ejection, microfluidics, micropatterning, nano dispensing, XFEL

## Abstract

Precise initiation and synchronization of chemical reactions in free space remain major challenges for time‐resolved studies in structural biology, chemistry, and materials science. Although modern probes provide femtosecond resolution, experimental access to early reaction dynamics is limited by the ability to trigger, mix, and deliver nano‐to‐picoliter liquid samples with deterministic spatial and temporal control. Existing liquid jets and drop‐on‐demand systems cannot reliably define reaction start times in flight or synchronize them to external pulse patterns. We present an acousto‐pneumatic droplet ejection platform that delivers single sub‐nanoliter droplets on‐demand with microsecond timing accuracy and reproducible trajectories, enabling deterministic one‐drop‐per‐trigger operation synchronized to external signals at repetition rates up to 100 Hz. Deterministic mid‐air droplet collisions define reaction initiation directly at the probe and are compatible with demanding pulse structures such as X‐ray free‐electron lasers. The programmable platform offers high‐resolution, time‐resolved experimentation, spanning ultrafast X‐ray studies and precision nanoliter dispensing.

## Graphical abstract

LiDIA is an on‐demand droplet‐in‐air platform that enables synchronized droplet collision, ultralow‐waste operation, and obstruction‐free sample delivery. By combining precise triggering with reliable free‐space dispensing, it provides a versatile route for rapid mixing and time‐resolved studies, with strong potential for applications in XFEL, cryo‐EM, and other dynamic analytical workflows.

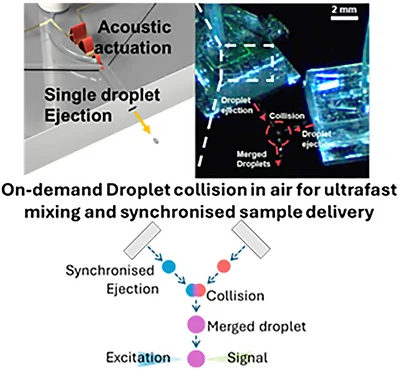

## 1 Introduction

Monitoring real‐time progression of chemical reactions with ultrahigh temporal resolution is essential across a wide range of fields including structural biology [1], materials science [2, 3, 4], and biomedical research [5]. Techniques such as X‐ray free‐electron lasers (XFELs) provide high‐intensity probe pulses capable of capturing transient intermediates in protein folding [1, 6, 7] or enzymatic reactions [5] with femtosecond resolution [1, 8, 9]. In these experiments, reactions are rapidly initiated and probed at defined time delays with ultrashort, high‐intensity X‐ray pulses, enabling time‐resolved structural measurements via diffraction before destruction. These same approaches are also adopted in materials science to investigate ultrafast and transient processes, such as nanoparticle nucleation or other structural transition events [2, 3, 4]. These techniques, however, impose stringent demands on sample delivery.

To access early reaction dynamics, reagents must be mixed and delivered into air or vacuum with precisely defined timing and synchronized to external pulse patterns [1, 8, 10, 11, 12, 13]. Conventional droplet microfluidic formats are poorly suited to this workflow because carrier oils and solid channel walls obstruct the X‐ray path and because mixing and injection are difficult to synchronize with high‐repetition‐rate probes.

Gas dynamic virtual nozzles (GDVNs) are widely used at XFEL facilities to deliver continuous microjets of sample into air or vacuum [12, 14, 15]. A coaxial gas sheath hydrodynamically focuses the liquid into a thin jet with a stable trajectory that is compatible with high repetition rates, and upstream mixers can be incorporated for reaction initiation [15]. However, continuous jets are difficult to phase‐lock to individual pulses and typically consume large volumes of often precious samples. Recent development of on‐demand dripping modes significantly improves hit rates but does not fully resolve the issue of sample waste [16].

Drop‐on‐demand (DOD) schemes reduce consumption by ejecting discrete droplets onto a translating tape (drop‐on‐tape) [13, 17]. This configuration allows spatial targeting, and mixing can be initiated by depositing droplets onto a sessile drop [18]. In such a configuration, mixing is driven by impact‐induced flows and typically requires on the order of 10^2^ ms for homogenization, with additional time delay for the tape to translate the droplet into the probing zone [18]. These delays limit access to the onset of the reaction, while polymer tapes introduce background scattering, and the droplet deposition methods are incompatible with vacuum operation.

Variations in droplet size, arrival time, or trajectory can compromise experimental resolution, reduce reproducibility, and dramatically increase sample consumption [11]. This has created a broader and growing demand for droplet ejection systems that offer sub‐nanoliter volume control, precise spatial targeting, and strict temporal determinism, ideally in an on‐demand and calibration‐free format that remains compatible with open environments and downstream instrumentation.

**FIGURE 1:**
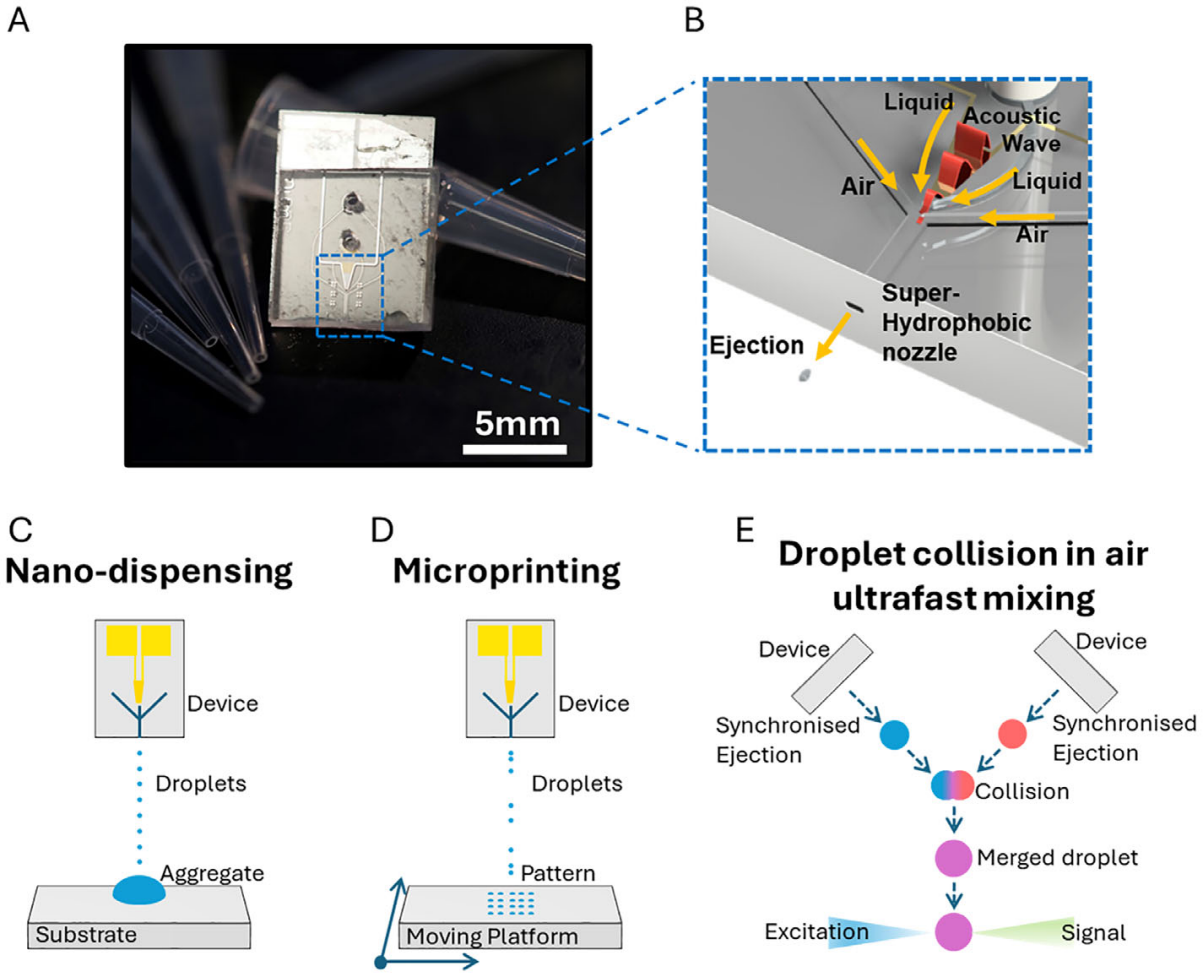
Overview of the LiDIA device and operating modes. (A) Photograph of LiDIA shown next to 10 µL micropipette tips for scale, (B) Schematic of the device operation. (C–E) Illustrations of key application configurations: (C) sub‐nanoliter dispensing, (D) high‐resolution microprinting, and (E) synchronized on‐demand droplet collision in air for ultrafast reagent mixing.

To overcome these constraints, we developed LiDIA (Liquid Droplet‐in‐Air), shown in Figure 1A, a compact acousto‐pneumatic platform for deterministic droplet generation and synchronized reaction initiation in free space. LiDIA combines the programmability of DOD systems with the directional stability and vacuum compatibility associated with GDVNs. As depicted in Figure 1B, a pulsed, focused surface acoustic wave (SAW) induces droplet formation within a gas‐filled microchannel. The droplet is subsequently entrained and accelerated by a co‐flowing gas stream and ejected through a superhydrophobic outlet into free space, such as a gas or vacuum environment.

While mature commercial liquid handling technologies such as Echo, iDOT, and BioDot platforms excel at automated well‐plate loading, liquid transfer, and substrate spotting, they are optimized for defined dispensing geometries rather than time‐controlled free‐space droplet delivery. Compared with these existing liquid‐handling technologies (Section S1 and Table S1), LiDIA provides robust one‐pulse/one‐drop control, improved spatial repeatability in free flight, precise ejection timing, and reduced constraints on ejection. Unlike thermal and piezoelectric inkjet systems, satellite droplets are avoided, and drop‐to‐drop variability is minimized [19]. While prior SAW‐based implementations have used engineered nozzles to demonstrate on‐demand droplet trains [20], deterministic single‐droplet ejection with defined trajectories has remained challenging. LiDIA delivers sub‐nanoliter droplets with low volume variation (CV< 5%), temporal accuracy below 80 µs, trajectory deviations under 5 µm, over 2 mm of travel, and programmable one‐pulse/one‐drop operation from single events to repetition rates of 100 Hz [11].

This level of spatiotemporal precision enables two LiDIA ejectors to be aligned and synchronized to generate colliding droplets directly at the probe. In‐flight impact produces rapid mixing within a few hundred microseconds [21, 22], providing earlier access to reaction dynamics than substrate‐based implementations while preserving efficient sample utilization. Although compatible with demanding pulse structures such as those of XFELs, the platform is not restricted to X‐ray studies.

Beyond ultrafast probe experiments, LiDIA's precise volume metering, spatial repeatability, and timing determinism support applications including mass spectrometry interfaces, rapid sample preparation for time‐resolved cryo‐electron microscopy [6, 23], nanoliter dispensing, and microdroplet arraying. Nanoliter dispensing and microdroplet arraying. In this work, we quantify droplet volume control, trajectory stability, and ejection timing through nanoliter‐scale metering (Figure 1C), droplet‐based micropatterning (Figure 1D), and synchronized in‐air droplet collisions (Figure 1E), establishing droplet‐in‐air microfluidics as a programmable platform for precision time‐resolved experimentation.

## 2 Working Principle

### 2.1 Device Configuration

**FIGURE 2:**
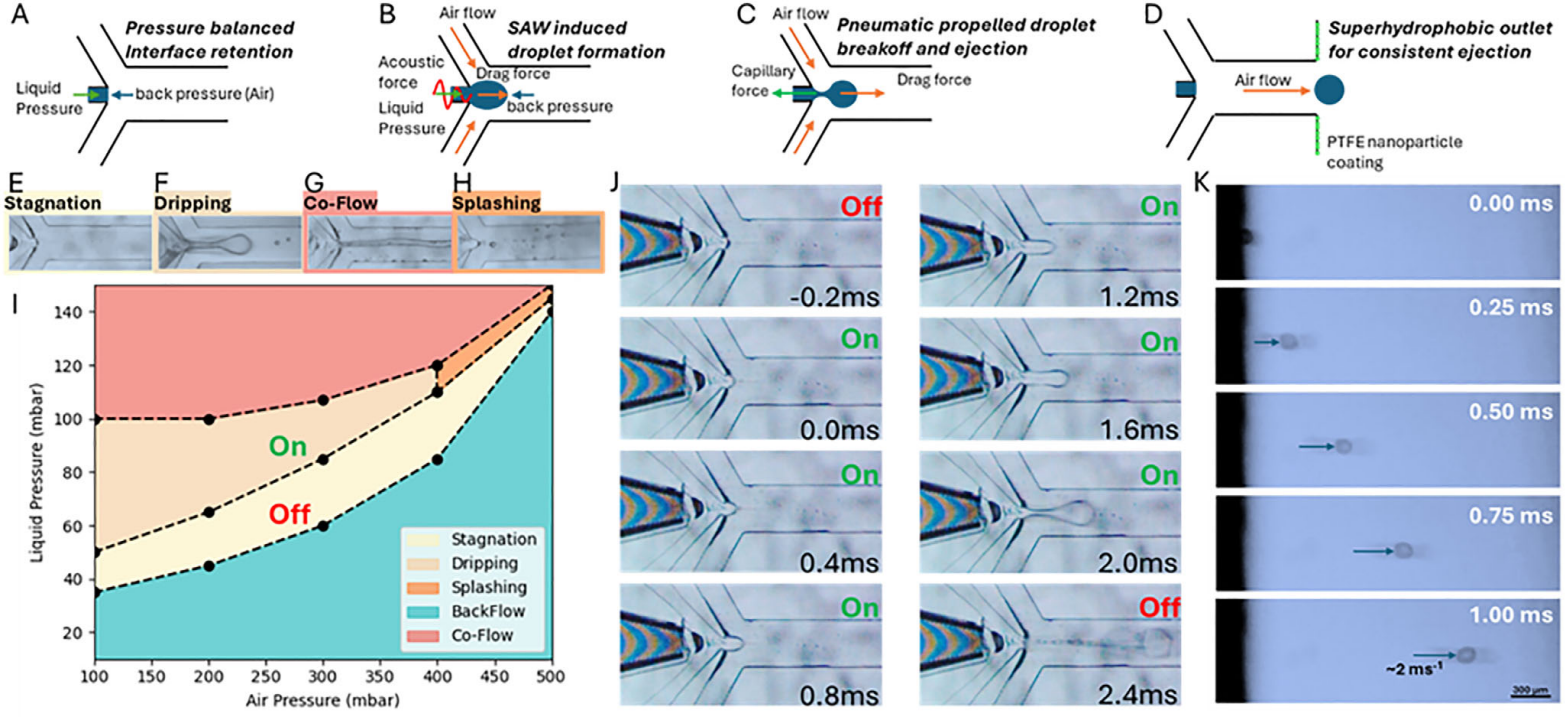
Working principle and operating regimes of LiDIA. (A–D) Illustration of LiDIA's working principle. (E–H) Images of the flow‐focusing junction under different flow regimes. (I) Flow‐regime map showing the relationship between system response and liquid/gas pressure settings. (J) Image series showing droplet formation initiated by acoustic actuation and subsequent break‐off driven by the air flow. (K) Image series showing a droplet exiting the superhydrophobic outlet and accelerating in the air stream.

The LiDIA device is constructed with a lithium niobate (LnNbO_3_) piezoelectric substrate, patterned with interdigitated transducers for producing focus surfacer acoustic waves, and a PDMS‐based microfluidic chip plasma‐bonded to the substrate, with the channel features aligned to the IDTs. The channel features a flow focusing junction [24] with a 60° inclination angle (Figure 2A). Liquid to be ejected is delivered to the chip by a pressure pump at a set pressure through PTFE capillary tubing to the middle channel of the chip, and air is delivered the same way to the side channels of the chip. During operation, the liquid pressure and gas pressure are set to a balanced condition that establishes a steady equilibrium in which the liquid‐air interface remains stationary and no droplets are produced. (Figure 2A). Droplets are produced by a short 191 MHz electrical pulse applied to the IDTs, which generates a SAW pulse that induces acoustic streaming [20] to momentarily translate the liquid front, thereby generating droplets (Figure 2B) that subsequently are carried downstream by the air stream's drag force (Figure 2C). The outlet is coated with PTFE particles to be superhydrophobic to prevent adhesion of the droplet and facilitate consistent ejection of the droplet (Figure 2D). Droplet ejection only happens when a pulse is provided, and only one droplet is ejected for every single pulse. The pulsing can be repeated at different rates to achieve different desired ejection rates, from a single ejection event to over 100 drops per second.

### 2.2 Fluid Pressure and Flow Regimes

To achieve on‐demand droplet generation and ejection, liquid and gas pressures are precisely balanced so the liquid‐air interface remains pinned at the nozzle without forming droplets (stagnation regime, Figure 2E). When an acoustic pulse is applied, the liquid pressure increases momentarily, the liquid interface is deformed, and droplets are generated in the dripping or co‐flow regime. When the pulse is switched off, the flow transitions back to the stagnation regime. The duration for which the system remains in the elevated flow regime is governed by the applied pulse width.

Maintaining this pressure balance between the gas and liquid regions is critical to control the droplet formation process. If the liquid pressure is too low relative to the gas pressure, the gas will push the liquid backward and intrude into the liquid channel (backflow regime). If the liquid pressure is too high, droplets will form continuously rather than on demand (dripping regime, Figure 2F). Further increasing the liquid pressure results in a continuous liquid stream flowing alongside the air stream (co‐flow regime, Figure 2G). In some cases where both liquid and air pressures are high, the advanced liquid front will not form a single droplet but instead shatters into multiple small droplets (Splashing regime, Figure 2H).

The required pressure balance depends directly on channel geometry, especially the downstream channel length (the distance from the droplet formation junction to the exit nozzle). Because air flows continuously, it experiences flow resistance, which creates backpressure at the liquid front. The strength of backpressure varies with the position of the interface. The liquid pressure must therefore be high enough to overcome both this backpressure and the adhesion between liquid and channel walls to deliver liquid to the junction, but not so high that droplets form automatically. Changes in channel length and geometry alter the air‐phase resistance and thus the backpressure. This pressure depends on many factors, including channel length on the chip and in the connections to the pumps, as well as surface adhesion forces, which makes theoretical calculation difficult and inefficient. We therefore studied the system's behavior empirically by exploring the system's response under different combinations of liquid and gas pressures and constructed a flow‐regime map to illustrate their relationship (Figure 2I). We concluded that to maintain stagnation, the gas pressure is typically two to three times the liquid pressure. Because the meniscus is maintained by the pressure balance between the gas and liquid streams rather than by gravity, the device can be operated in any orientation, which also defines the droplet ejection direction.

During operation, the liquid pressure is set near the upper boundary of the stagnation regime to promote rapid interface recovery after droplet ejection. This is especially important when high ejection rates are required, as the liquid consumed in the previous ejection event needs to be replenished before the next ejection in order to maintain liquid level for consistent ejection behavior.

### 2.3 Surface Acoustic Wave (SAW) Actuation Pulse Parameters and Effects

Unlike conventional piezoelectric drop‐on‐demand systems, which mechanically deform the fluid chamber to drive fluid motion, LiDIA uses surface acoustic waves to directly impart momentum to the liquid itself. As the focused surface acoustic wave enters the liquid, a portion of the acoustic energy is attenuated and transferred to the fluid, producing a local body force. This force perturbs the pressure‐balanced liquid/gas interface, translating the liquid front forward and momentarily shifting the operating point on the regime map. The acoustic actuation effectively pumps the liquid, increasing its flow rate; the pulse amplitude therefore determines how far the operating point moves along the y‐axis of the map. The ideal pulse energy moves the system just into the dripping regime, which yields the most consistent droplets. Excessive power can push the system into coflowing or even splashing, which prevents proper droplet formation.

Pulse width controls how long the system stays in that regime. In other words, it sets how long we pump. For strict 1‐pulse‐1‐drop control, the pumped volume should match the volume of a single droplet. If the pulse is too long, excess liquid produces more than one droplet per pulse or even a transient co‐flow, which is undesirable. Longer pulses can also cause heating, cavitation, or damage to the crystal substrate. We found an optimal combination of amplitude and duration to be 32 dBm and 2 ms, which corresponds to a pulse energy of approximately 3.17 mJ.

When the pulse is applied, the liquid front overcomes the air backpressure and wall adhesion and begins to form a droplet (Movie S1). As the droplet grows, the drag force increases. When the droplet reaches a critical diameter (150 µm in our device), the drag force exceeds the capillary force (surface tension) connecting the spherical front to the bulk liquid and the adhesion to the walls. In doing so, the front detaches and forms a droplet that is then carried downstream by the air stream. A visual representation of the process is displayed in Figure 2J.

### 2.4 Superhydrophobic Outlet for Clean Ejection

Downstream, at the exit, the outer surface of the device is rendered superhydrophobic, which ensures full dewetting and prevents droplets from clinging to the outer surface (Figure 2D). Without this coating, exiting droplets tend to wet the nozzle tip, which could severely skew the ejection trajectory and cause liquid accumulation at the exit. To coat the outlet, we developed a simple method inspired by how dust adheres to PDMS: the outer surface is pressed against PTFE nanoparticles (Figure S2), then excess powder is removed with air and water jets. Compared with previous approaches that use chemical treatments or spray coatings [25], this method is simpler and can be reapplied easily. It dramatically improves surface hydrophobicity and is essential for reliable ejection. Figure 2K and Movie S2 show a droplet ejected from the superhydrophobic outlet surface.

### 2.5 Air Damper Stabilizing the Droplet Interface

**FIGURE 3:**
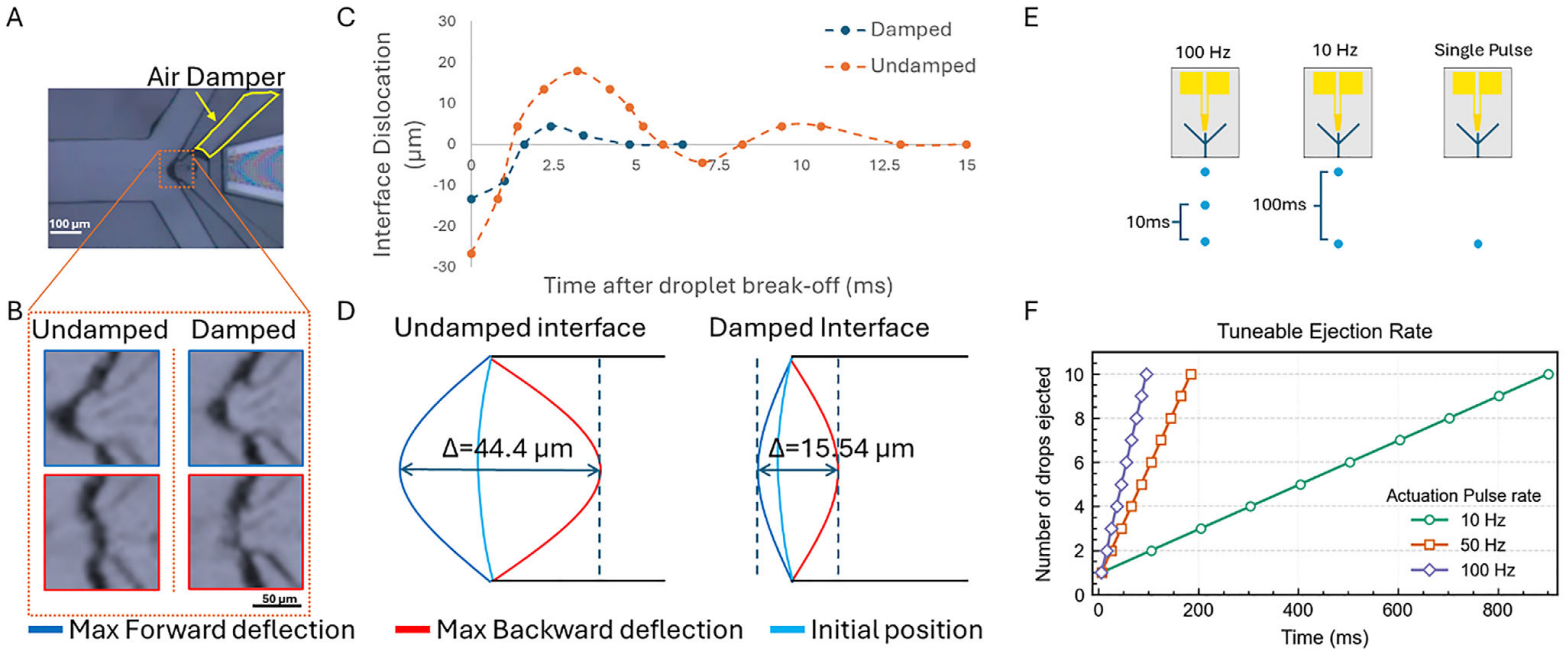
Air damper stabilizes the interface and enables rate control. (A) Image of the channel highlighting the air damper. (B) Images of the fluid interface at the maximum forward (blue) and backward (red) deflections after droplet break‐off. (C) Fluid‐interface position vs. time for damped and undamped operation. (D) Schematic comparing the initial interface position and the maximum forward/backward deflections for damped and undamped operation. (E) Schematic illustrating tuning of droplet ejection rate by adjusting the actuation frequency. (F) Cumulative droplet count vs. time at actuation frequencies of 10, 50, and 100 Hz.

Controlling the stability of the interface and its recovery after each ejection event is crucial to enable on‐demand ejection and avoid satellite droplets. This also shortens the recovery time between events, enabling higher‐frequency operation. To achieve this, in addition to the pressure‐balanced junction and SAW actuation, the device also incorporates an air bubble in the liquid channel, which acts as a hydraulic capacitor (Figure 3A) that dampens pressure fluctuations from the droplet breakoff event and thus reduces interface oscillation duration and amplitude (Figure 3B). Without this capacitor, the interface displacement can be nearly 3 times larger (Figure 3C,D) at 44.40 µm without and 15.5 µm with the damper, and occasionally produces unwanted secondary droplets instead of the desired single droplet ejection per pulse due to the large interface deflection. The air bubble damper also reduces the settling time of the interface after break‐off. Without the damper, the fluid interface takes longer to stabilize, taking 13 ms, as compared to the case with damping, which only takes less than 5 ms (Figure 3C).

### 2.6 Tunable Ejection Rate via Pulse Timing

LiDIA's ability to produce exactly one droplet per actuation pulse enables deterministic control over the droplet ejection rate by adjusting the actuation rate. Since droplets are formed only when a SAW pulse is applied, the device remains idle between actuations with no spontaneous ejection, resulting in a one‐to‐one mapping between each actuation cycle and a droplet ejection event. As illustrated in Figure 3E, ejection rate can be directly tuned by modifying the timing of SAW pulse delivery: an actuation rate of 100 Hz yields droplets at 10 ms intervals, 10 Hz gives 100 ms droplet spacing, and a single pulse ejects a single droplet. This concept is experimentally validated in Figure 3F and Movie S3, where the cumulative number of ejected droplets is plotted over time for representative actuation rates of 10, 50, and 100 Hz. In each case, the ejection proceeds linearly and precisely matches the expected rate, confirming consistent and reliable pulse‐to‐droplet conversion across a broad operational range.

### 2.7 Handling Viscous Liquids

To investigate the effect of liquid viscosity on LiDIA's operation, aqueous glycerol solutions of 0, 15, and 30 wt.% concentration, which correspond to dynamic viscosities of 1.0, 1.5, and 2.5cP [26], were tested. LiDIA was able to eject liquids within this range. As shown in Table S2, the SAW pulse power and pulse length required for droplet formation remained identical across this viscosity range. This is because, at higher viscosities, the increased resistance to fluid motion is counteracted by the increased acoustic attenuation [27]. In contrast, the required liquid pressure setting increased linearly with viscosity from 60 mbar for 1cP to 65 mbar for 1.5cP, and to 75 mbar for 2.5cP, to compensate for the increased hydraulic resistance. Detailed theoretical explanations of these viscosity‐dependent effects are provided in Section S2.

### 2.8 Droplet Volume and Consistency

Being able to maintain the volume of the ejected drop is necessary for precise sample handling and dosage control in analytical applications. In our system, the droplet size is primarily governed by channel dimensions. As described above, droplet detachment occurs when the aerodynamic drag exerted by the air stream exceeds the forces retaining the droplet. These include the adhesion force between the droplet and the channel walls (from contact with the top and bottom surfaces), and the capillary force connecting the droplet to the liquid within the channel. The capillary force is set by the liquid channel's geometry and remains constant, and the adhesion force increases as the droplet grows due to the enlarged contact with the channel walls.

As the droplet diameter increases, the aerodynamic drag acting on it also rises. This is due not only to the increase in projected frontal area but also to the greater obstruction it poses to the surrounding flow. For a stationary droplet inside a tube with air moving around it, this can be expressed as Equation (1) [28].

(1) $$F_{drag}=\frac{1}{2}\rho v^{2}AC_{d}K$$

where ρ is the density of air, *v* is the velocity of the incoming airstream, *A* is the projected frontal area, *C_d_* is the drag coefficient, and *K* is the drag coefficient modification factor for confined flow. The Haberman and Sayre wall correction factor, expressed as Equation (2) [28], which was developed for the motion of fluid spheres in moving liquids inside cylindrical tubes in the low Reynolds number regime, is used as a close approximation of our system.

(2) $$K=\frac{1-\frac{2}{3}\lambda^{2}-0.20217\lambda^{5}}{1-2.1050\lambda +2.0865\lambda^{3}-1.7068\lambda^{5}+0.72603\lambda^{6}}$$

where *λ* is the ratio of the radii of the droplet and the channel width. Accordingly, the drag force acting on the droplet increases nonlinearly as the droplet occupies more of the channel. The droplet size is therefore primarily determined by the channel dimension. We have observed that droplet breakoff occurs once it grows to approximately 60% of the width of the channel.

An optimal set of operating parameters was empirically identified: 200 mbar air pressure, 60 mbar liquid pressure, and a 2 ms pulse at 32 dBm amplitude with a 191 MHz actuation frequency. Under these conditions, droplet size was measured at the moment of ejection from the device by manually analyzing high‐speed video recordings frame‐by‐frame. The average size of ejected droplets is found to be 113 µm in diameter, with a standard deviation of 1.74 µm. This corresponds to an average droplet volume of 756 pl, with a CV of 4.7%. These droplets are ejected with a typical exit velocity of approximately 2 m/s (Figure 2K).

While the current device was rigorously optimized around these specific parameters to enable the synchronized mid‐air collisions demonstrated later in this study, the platform's output can be modulated. According to the governing physical principles, droplet ejection velocity can be adjusted by tuning the carrier gas pressure, while the baseline droplet volume can be altered by scaling the microfluidic channel dimensions.

### 2.9 Non‐Contact Nano Dispensing

With its highly consistent droplet volume, this platform can be utilized for high‐precision, nano‐volume dispensing by leveraging its one‐pulse/one‐drop on‐demand ejection, where each actuation pulse produces exactly one droplet of a known volume *V_drop_* =  756 *pL*. The total delivered volume can therefore be set deterministically by the pulse count (Equation 3).

(3) $$V_{\mathrm{dispense}}=N_{\mathrm{pulse}}\times V_{\mathrm{drop}}$$

**FIGURE 4:**
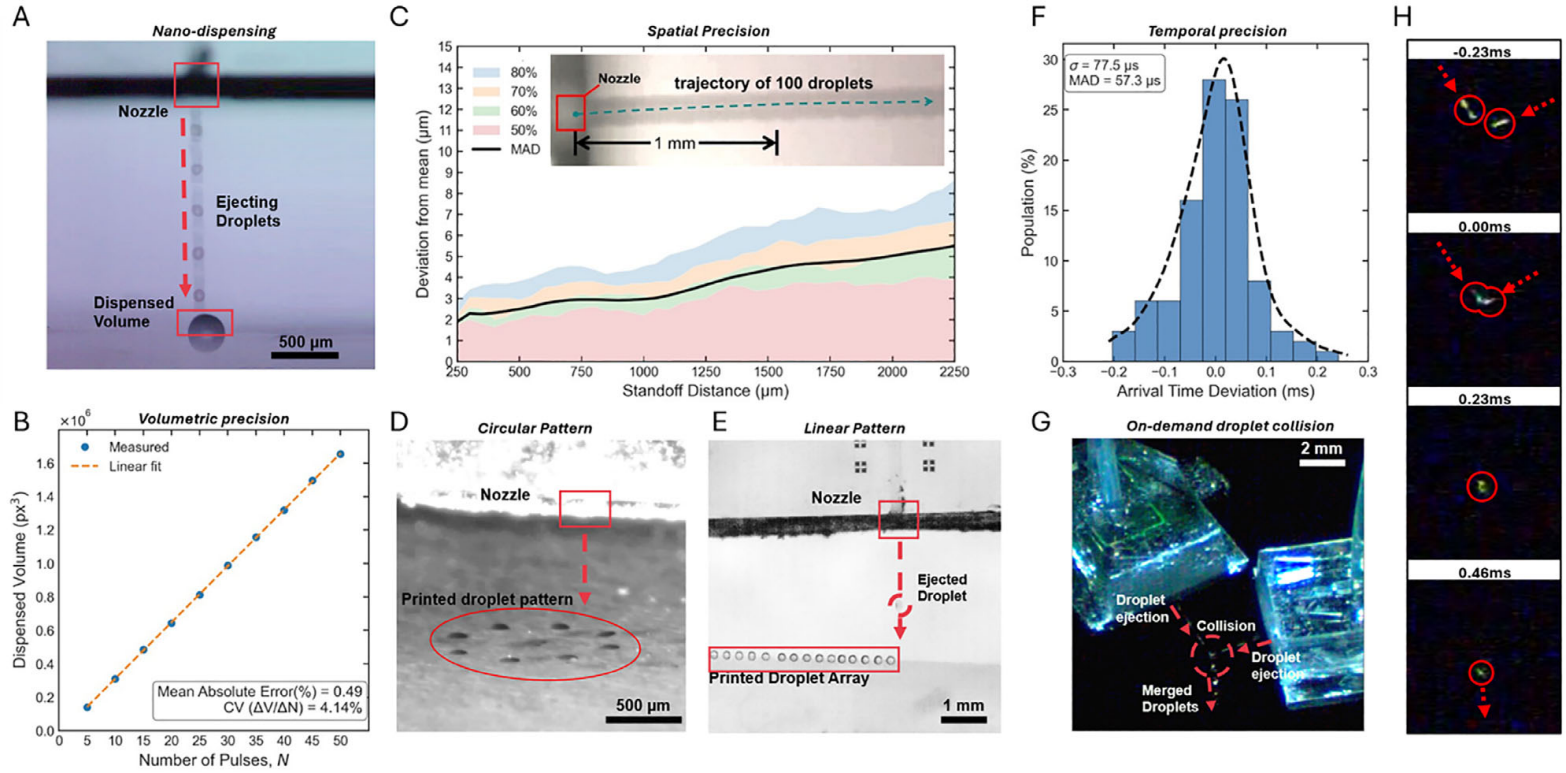
Characterization and demonstration of LiDIA's volumetric and spatial precision. (A,B) Volumetric accuracy characterized by deposited volume from cumulative droplet ejection: (A) overlaid high‐speed frames and resulting deposited volume on a surface and (B) dispensed volume vs. actuation pulse count, demonstrating precise nano‐dispensing. (C–E) Spatial precision characterized by trajectory repeatability: (C) droplet trajectory deviation over a 2250‐µm travel distance (inset, stacked overlay of 100 trajectories; mean absolute deviation, MAD) and demonstrations of microprinting by targeted droplet placement along (D) a linear path and (E) a circular path. (F–H) Temporal precision characterized by ejection timing: (F) histogram of droplet arrival‐time deviation and an on‐demand droplet‐collision demonstration in air, including (G) the experimental configuration and (H) a high‐speed image sequence of the collision process for synchronized ultrafast mixing.

This enables sub‐nanoliter accuracy without the need for real‐time feedback. For instance, 12 pulses will yield 9.07 nL, and 24 pulses yield 18.14 nL. As demonstrated in Figure 4A,B and Movie S4, the volume of dispensed liquid is linearly proportional to the number of pulses delivered, satisfying the expected relationship between dispense volume and number of pulses.

By comparing the change in dispensed liquid volume (ΔV) and the number of pulses (ΔN), we calculate a 4.14% coefficient of variation of the volume, which is consistent with the droplet volume variation characterization as detailed above.

This directly benefits workflows that require stringent volumetric control and minimal sample loss, including: miniaturized biochemical assays in wells or microreactors (e.g., enzyme kinetics, immunoassays); plate‐based genomics workflows such as qPCR/digital PCR master‐mix preparation and titration; single‐cell protocols that demand picolitre‐to‐nanoliter dosing with high fidelity; high‐throughput drug screening with combinatorial additions on microtiter plates; non‐contact transfers for clinical or environmental sample preparation that reduce carryover risk; precision deposition for materials and biosensing (e.g., nanoparticles, functional inks); and controlled nanodroplet delivery for crystallization experiments.

### 2.10 Spatial Accuracy

Another important aspect is the ability to deliver the liquid to the desired position accurately. We analyzed the repeatability of the ejected droplet's trajectories by tracking each droplet's position after exiting the device. The results, shown in Figure 4C, reveal that the trajectory spread of ejected droplets increases progressively with travelled distance. At short travel distances (500 µm), droplets exhibit minimal deviation from the mean trajectory, with the 50% band tightly clustered and a mean absolute deviation (MAD) approximately 2.5 µm. As the droplets travel further, the width of the percentile bands expands, and the MAD gradually rises, approaching 3 µm at a travel distance of 1000 µm and to approximately 5 µm at a travel distance of 2000 µm. Note that even at a long travel distance of 2000 µm, 80% of droplets fall within 7.5 µm. The absence of abrupt jumps or non‐linear growth in spread suggests a stable and repeatable ejection mechanism. For comparison, a previous study reported commercial acoustic liquid handling system (Echo 550) has average radial displacements between 30 and 50 µm for 2.5 nL droplets [29]. This demonstrates LiDIA's high spatial precision and underscores its suitability for precision droplet delivery applications.

### 2.11 Microprinting

Harnessing the high spatial repeatability of the system, we showcase its potential applicability for microprinting applications. Through synchronized control of droplet ejection and substrate translation, the device can be utilized to deposit liquid samples to a desired precise location. By coordinating single‐pulse actuation with the motion of a motorized stage, droplets are deposited at programmed spatial coordinates, enabling one‐pulse/one‐drop delivery at user‐defined discrete points. In Figure 4D,E and Movies S5 and S6, we showcase the device for depositing droplets on various points along a linear path and along a circular path, respectively, demonstrating the device's potential utility with its precise spatial control and synchronizability.

This capability is well suited for applications such as DNA and protein microarray spotting, surface‐based biochemical assays, and the localized deposition of functional materials for sensor and microelectronics fabrication. In addition, the platform's spatial precision and deterministic actuation make it compatible with drop‐on‐tape sample delivery systems used in time‐resolved crystallography at XFEL facilities, where substrate droplets must be accurately deposited onto crystal‐containing droplets with tightly controlled timing to initiate reactions immediately prior to X‐ray exposure [17, 18]. The ability to dispense small volumes without satellite formation and with low jitter supports reaction initiation with reduced sample consumption and improved temporal resolution. The platform is readily integrated with automated or scanning systems. A single controller can regulate both stage motion and droplet actuation, allowing placement to be defined by coordinate lists and local dosing by pulse count.

### 2.12 Temporal Precision

Another important feature of our device is the precise control of the timing of ejection events. To evaluate the temporal stability of the ejection process, we analyzed high‐speed video recordings of successive droplet ejections. For each frame, the position of the ejected droplet was recorded, and a velocity profile was fitted to determine the time each droplet reached a set position (500 µm downstream from the device exit nozzle). The timing jitter was then calculated using the root mean square deviation from the mean arrival time (Equation 4).

(4) $$Jitter=\sqrt{\frac{1}{N}\sum_{i=1}^{n}{\left(t_{i}-t_{\mu }\right)}^{2}}$$

The *jitter*, shown in Figure 4F, was found to be 77.5 µs. Consider an ejection period of 10 ms (for 100 drops per second operation); this corresponds to a timing variation of less than 0.8%. These results show the system's sub‐millisecond temporal precision, confirming its suitability for synchronized operation with high‐speed analytical techniques and other time‐sensitive processes.

### 2.13 Drop‐On‐Drop Reaction Initiation

The platform's spatial precision, volumetric accuracy, and deterministic ejection capabilities are favorable for drop‐on‐tape sample delivery strategies used in time‐resolved crystallography at XFEL facilities [18]. To demonstrate this, we performed a drop‐on‐drop reaction initiation experiment using calcium carbonate precipitation as a model reaction. A LiDIA device was used to eject a single droplet of calcium chloride solution (0.5 M) into a sessile drop of 0.5 M Sodium carbonate. Upon mixing, calcium ions react with carbonates and form calcium carbonates (CaCO_3_), producing visible precipitates within the sessile droplet. The process was recorded using high‐speed videography under transmitted light illumination, presented in Movie S7. Further details of the set‐up are provided in the Supporting Information.

Upon injection of the CaCl_2_ droplet, a distinct vortex‐like pattern formed within the sessile droplet. This feature is attributed to the refractive‐index contrast between the two concentrated salt solutions and mixing due to the impact‐induced flow within the sessile droplet. The vortex‐like pattern dispersed and largely disappeared after approximately 30 ms, indicating completion of the mixing process. Following the dissipation of the initial refractive‐index pattern, small CaCO_3_ precipitate particles formed and were observed to be swirling within the sessile droplet, driven by the residual internal circulation generated by the droplet impact. The precipitate particles then gradually increased in size over time, confirming the continued progression of the precipitation reaction after the initial mixing event.

This validates the platform's utility for precisely timed, volume‐controlled reaction initiation.

### 2.14 Ultrafast Reagent Mixing Through On‐Demand in Air Droplet Collision

Building on the high spatiotemporal precision of LiDIA, we demonstrate the platform's potential capacity for precise reaction control by in‐air droplet collision. In this experiment, we aligned two droplet ejection devices and synchronized their actuation timing to eject a single droplet from each device simultaneously to collide them in air, which, through this process, merged into a single droplet, as shown in Figure 4G,H and Movie S8. This level of control is enabled by the system's pulse‐based synchronization and highly repeatable ejection trajectory.

Mid‐air droplet collision has been shown to achieve ultrafast mixing, with prior studies reporting mixing times on the order of 100 ms under a continuous droplet jetting configuration [22]. In contrast, our platform offers an on‐demand alternative producing discrete collisions at user‐specified times and locations. This creates new opportunities for coupling reaction initiation with downstream time‐resolved characterization techniques, such as Raman spectroscopy, mass spectroscopy, or serial femtosecond crystallography (SFX) at XFEL facilities. By triggering reactions through collision‐based mixing and synchronizing the ejection event with the arrival of probe pulses, our system could enable highly time‐resolved kinetic studies.

Beyond optical and X‐ray studies, this capability could also be extended to sample preparation for time‐resolved cryo‐electron microscopy (cryo‐TEM). For example, biochemical reactions could be initiated in mid‐air via droplet collision and immediately deposited onto a TEM grid with a synchronized cryo‐quenching action. This shows strong potential in minimizing processing time and enhancing temporal resolution, where current techniques struggle to prepare samples below 100 ms [6, 23]. The one‐pulse/one‐drop control and high spatiotemporal precision of the platform ensure that such events can be precisely timed and repeated, offering improved control over sample preparation and data acquisition. Importantly, since mixing occurs entirely in free space and without contact with device surfaces or substrates, the approach also minimizes the risk of cross‐contamination. This substrate‐free reaction initiation method adds a new layer of flexibility to high‐precision, temporally coordinated experimental workflows. In the current implementation, each LiDIA device dispenses a single liquid solution at a time. However, the platform can be readily extended to manage multiple samples sequentially. The liquid can be retracted by releasing the applied driving pressure to allow for reservoir exchange, or a multi‐inlet microfluidic manifold can be integrated upstream for automated switching. Thus, while the present study focuses on single‐solution dispensing from each nozzle, the platform possesses the inherent scalability to handle multiplexed liquid resources.

## 3 Conclusion

LIDIA is a hybrid acousto‐pneumatic on‐demand droplet ejection system that achieves deterministic, one‐to‐one droplet generation with high spatial and temporal precision. By combining surface acoustic wave (SAW)‐triggered nucleation with pneumatic actuation and a superhydrophobic nozzle, LIDIA consistently produces single droplets on demand with sub‐nanoliter volume, minimal variation (<5% CV), <5 µm trajectory repeatability, and <80 µs ejection timing jitter. These characteristics address long‐standing limitations in conventional inkjet, electrohydrodynamic, and valve‐based dispensing technologies, particularly in applications where timing, volume fidelity, and droplet stability are critical.

Unlike commercial dispensers optimized either for static well deposition or to prioritize high‐throughput delivery, LiDIA operates in an event‐based regime, delivering exactly one droplet per trigger with direct synchronization to external signals. This deterministic pulse‐to‐drop mapping enables volume quantization by pulse count and simplifies integration into time‐sensitive workflows without complex calibration procedures. The compact format and open‐air compatibility further distinguish LiDIA from continuous jet systems designed primarily for steady‐state operation.

This operational mode enables experimental capabilities not accessible with existing technologies. Deterministic droplet timing and trajectory control allow synchronized in‐air droplet collisions for rapid reaction initiation and precise sample positioning. Such control is compatible with demanding probe pulse structures, including XFEL‐based serial crystallography, while also supporting nano‐dispensing, micro arraying, and other precision liquid‐handling applications requiring sub‐nanoliter accuracy.

By enabling event‐based synchronized droplet generation in free space, LiDIA establishes droplet‐in‐air microfluidics as a programmable approach to precision‐controlled reaction initiation and time‐resolved experimentation across biological, chemical, and physical sciences.

## 4 Materials and Methods

### 4.1 Experimental Setup

All experiments were performed using deionized (DI) water as the working fluid. Illumination was provided by a Thorlabs SOLIS‐1D collimated LED, and droplet motion was imaged through a Nikon Eclipse Ci microscope coupled to an InfinityProbe C‐mount video Microscope (Infinity Photo‐Optical) onto a Chronos 2.1‐HD high‐speed camera (Kron Technologies) to capture droplet formation, detachment, and free‐flight dynamics. Both the liquid sample and pneumatic airflow were supplied at controlled pressures using a Fluigent MFCS‐EZ pressure control system, enabling independent and stable regulation of the liquid feed and airflow.

### 4.2 SAW Actuation Circuit

The interdigital transducers (IDTs) were driven by a Rohde & Schwarz SMC100A RF signal generator. The output was amplified using a Mini‐Circuits LZY22+ power amplifier (43 dB gain) and routed through a JFW 50S‐1872 AP2T reflective solid‐state RF switch, controlled by an Arduino Nano microcontroller. The RF switch gated the signal to deliver precisely timed bursts for one‐drop‐per‐pulse operation.

For synchronized dual‐device droplet ejection (in‐air collision experiments), the amplifier output was fed into a Mini‐Circuits ZA2CS‐600‐10W‐S RF splitter to drive two identical IDT devices simultaneously. A 5‐axis micro positioner is used for adjusting alignment between the two devices.

### 4.3 IDT Fabrication

The droplet ejection device was built on a 128° Y‐cut lithium niobate substrate, chosen for its high electromechanical coupling efficiency in SAW applications. The interdigital transducer layout was designed in KLayout and patterned onto the LiNbO_3_ wafer using maskless photolithography (Heidelberg MLA‐150). After spin‐coating and soft‐baking a positive photoresist, the pattern was exposed and developed. Electron‐beam evaporation deposited a tri‐layer metal stack: 10 nm chromium (adhesion layer), 180 nm aluminum (conductive layer), and 10 nm gold (protection layer). A lift‐off process removed excess material, leaving the IDT pattern. A thin SiO_2_ passivation layer was deposited by electron‐beam evaporation to protect the electrodes and improve bonding strength to PDMS. The finger spacing of the IDTs is 20 µm, resulting in a resonant frequency at around 190 MHz.

### 4.4 Microfluidic Device Fabrication

The microfluidic channel was fabricated using soft lithography. A master mold was prepared by spin‐coating and photopatterning SU‐8 photoresist to define the channel geometry. Sylgard‐184 PDMS elastomer and curing agent mix (10:1 ratio) was cast over the mold, cured on a hotplate at 60°C for 6 h, peeled, and trimmed to the desired size. The PDMS channel was aligned to the IDT region using a micro‐positioner and permanently bonded to the substrate via oxygen plasma treatment.

### 4.5 Surface Treatment

The microfluidic channel was treated with a fluorocarbon compound (Fussode COAT) to increase hydrophobicity. The nozzle outlet was rendered superhydrophobic by pressing the outlet surface into a powder bed of PTFE nanoparticles (Nanografi, 260 nm diameter) and then blowing off the excess powder with a lens cleaner.

### 4.6 Data Analysis

High‐speed videos of droplet ejection were recorded with the Chronos 2.1‐HD camera. Droplet size, position, and trajectory were analyzed using ImageJ and MATLAB. Custom MATLAB scripts were used to extract quantitative metrics including droplet volume variation, trajectory repeatability, ejection rate, timing jitter, and damping behavior. Data plots were generated in Python using Matplotlib.

## Author Contributions

Conceptualization: Hao Deng and Tuncay Alan. Methodology: Hao Deng and Tuncay Alan. Investigation: Hao Deng. Visualization: Hao Deng. Supervision: Tuncay Alan and Jacek J. Jasieniak. Writing – original draft: Hao Deng and Tuncay Alan. Writing – review and editing: Hao Deng, Tuncay Alan, and Jacek J. Jasieniak.

## Funding

This work was supported by the Australian Research Council Discovery Project Scheme DP260103767; Australia's Economic Accelerator Program AEA 240300119.

## Conflicts of Interest

The authors declare no conflicts of interest.

## Supporting information




**Supporting File 1**: smll74957‐sup‐0001‐SuppMat.docx.

**Supporting File 2**: smll74957‐sup‐0002‐MovieS1‐S8.zip.

## Data Availability

The data that support the findings of this study are available from the corresponding author upon reasonable request.
