# Supplementary material for: Precisely Timed On‐Demand Droplet Ejection and Collision in Free Space for Controlled Reaction Initiation
Source: Small. 2026 Aug 5;22(53):e74957. doi: 10.1002/smll.74957 (PMC13592412; doi:10.1002/smll.74957)
Supplement: Supplementary file 1 [file SMLL-22-e74957-s002.docx]

Supplementary Information

Precisely timed on-demand droplet ejection and collision in free space for controlled reaction initiation

Hao Deng, Jacek J, Jasieniak, and Tuncay Alan*

Supplementary Movie S1.

High-speed video showing the droplet formation process at the nozzle upon actuation by a short surface acoustic wave (SAW) pulse.

Supplementary Movie S2.

High-speed video showing a formed droplet exiting the device from the superhydrophobic outlet.

Supplementary Movie S3

High-speed video demonstrates droplet ejection at varying ejection rates of 100, 50, and 10 droplets per second.

Supplementary Movie S4

Video demonstrating the use of the device for precise on-demand nano volume liquid dispensing.

Supplementary Movie S5

Video demonstrates the use of the device for microprinting by sequential on-demand droplet deposition onto a moving substrate to form a linear droplet array.

Supplementary Movie S6

Video demonstrates the use of the device for microprinting by sequential on-demand droplet deposition onto a moving substrate to form a circular droplet array.

Supplementary Movie S7

Video demonstrates drop-on-drop reaction initiation of calcium carbonate formation.

Supplementary Movie S8

Video demonstrates on-demand in-flight droplet collision achieved by two devices aligned and operated in synchrony to eject droplets simultaneously.

**S1. Comparison of LiDIA with existing droplet delivery and liquid handling platforms**

Existing commercial droplet dispensing platforms, such as Echo, iDOT, BioDot, MANTIS, and piezoelectric microdispensers, are mainly designed for reagent transfer, well-plate loading, or surface deposition. Their performance is therefore typically evaluated by dispensed volume, volumetric precision, throughput, and substrate spotting accuracy.

LiDIA is designed instead for on-demand free-space delivery of individual droplets. Its key performance metrics are therefore droplet trajectory repeatability, trigger-to-ejection timing, and compatibility with synchronized droplet interactions. LiDIA achieves a trajectory spread below 5 µm over a 2 mm flight distance and an ejection timing accuracy below 80 µs, comparing favorably with reported spatial and temporal precision values for existing systems, as summarized in **Table S1**.

Unlike many commercial platforms, which are constrained to fixed upward or downward dispensing geometries, LiDIA stabilizes the liquid front through controlled liquid and gas pressures and transports droplets using a co-flowing gas stream. This reduces orientation constraints and enables free-space droplet delivery. The gas-assisted delivery principle is conceptually related to Gas Dynamic Virtual Nozzle (GDVN) injectors, suggesting that LiDIA is adaptable for vacuum-compatible sample delivery while retaining on-demand actuation, low sample consumption, and precise timing control.

**Table S1. Comparison of LiDIA with representative droplet delivery and liquid handling technologies**

| System | Technology | Droplet Volume | Spatial Precision | Temporal Precision | Ejection Direction | Vacuum Compatibility |
| --- | --- | --- | --- | --- | --- | --- |
| LiDIA (This work) | Acousto-pneumatic | 0.756 nl | <5 µm | <80µs | Unlimited | Possible |
| Microdrop MD-K-130^[1]^ | Piezoelectric micro-dispenser | 20-180 nl | 25 µm | Not reported | Downward | Not Compatible |
| Microdrop AD-KH-501^[2]^ | Piezoelectric microdrop generator | 0.421 nl | Not reported | 700µs | Downward | Not Compatible |
| Labcyte Echo 550^[3]^ | Acoustic droplet ejection | 2.5-50 nl | 30-50µm | Not reported | Upward | Not Compatible |
| Dispendix I.DOT^[4]^ | Positive pressure control | 8-50 nl | Not Reported | Not reported | Downward | Not Compatible |
| Formulatrix MANTIS^[5]^ | Micro diaphragm pump | >0.1µl | Not reported | Not reported | Downward | Not Compatible |
| Epson Stylus SX235W^[1]^ | Inkjet printer | 4 pl | 13 µm | Not reported | Downward | Not Compatible |
| Triggered dripping-mode GDVN^[6]^ | Piezo-triggered gas-dynamic virtual nozzle | 18.5 or 70 pl | 11 µm | <1000µs | Unlimited | Compatible |

**S2. Effect of viscosity to system parameters**

The liquid’s resistance to motion can be expressed through the viscous stress, using Equation S1 for Newtonian fluids.

$\sigma=\mu\frac{du}{dy}$	(S1)

where σ is the shear stress, μ is the dynamic viscosity, and du/dy is the velocity gradient. This term scales linearly with viscosity.

At the same time, the viscous attenuation of the acoustic wave, expressed using the acoustic attenuation per wavelength (Equation S2).

$\beta\lambda=\frac{4\pi\mu\omega}{3\rho c^{2}}$	(S2)

where β is the acoustic attenuation coefficient, λ is the acoustic wavelength, ω is the angular frequency, ρ is the liquid density, and c is the speed of sound in the liquid. For a fixed acoustic frequency, and assuming ρ and c do not vary significantly over the tested glycerol concentrations, this attenuation term also scales linearly with viscosity.

These two viscosity-dependent effects counteract each other,^[7]^ explaining why the required SAW pulse power remained independent of viscosity in our experiments.

To maintain operation, the liquid pressure setting had to be adjusted. For a channel with rectangular cross-section, the hydraulic resistance can be calculated using Equation S3.^[8]^

$R_{H}=\frac{12\mu L}{wh^{3}(1-0.63\frac{h}{w})}$	(S3)

where R_H_ is the hydraulic resistance, L is the channel length, and w and h are the channel’s width and height respectively. For a fixed channel geometry, this scales linearly to viscosity of the liquid, directly matching the observed linear increase of the required liquid pressure setting.

**Table S2.** Required liquid pressure setting and actuation power for liquid with different the dynamic viscosities:

| **Glycerol concentration** | **Dynamic Viscosity** | **Liquid pressure setting** | **Actuation power** |
| --- | --- | --- | --- |
| 0 wt% | 1.0 cP | 60 mbar | 32 dBm |
| 15 wt% | 1.5 cP | 65mbar | 32 dBm |
| 30 wt% | 2.5 cP | 75mbar | 32 dBm |

**S3. Drop-on-drop reaction initiation demonstration**

The platform’s spatial precision, volumetric accuracy, and deterministic ejection event also make it highly compatible with drop-on-tape sample delivery strategies used in time-resolved crystallography at XFEL facilities. In these approaches, a reagent droplet must be accurately deposited onto a pre-positioned sample droplet containing crystals, with tightly controlled timing to initiate the reaction shortly before X-ray exposure. LiDIA provides a direct route to this mode of operation by enabling single, sub-nanoliter droplets to be precisely delivered on demand to a defined location and at a defined time.

To demonstrate this capability, we performed a localized drop-on-drop reaction initiation experiment using calcium carbonate precipitation as a model reaction. A 0.5 µL sessile droplet of 0.5 M sodium carbonate (Na_2_CO_3_) was first placed on the substrate using a micropipette. LiDIA was then aimed at the sessile droplet, and a single 0.75 nL droplet of 0.5 M calcium chloride (CaCl_2_) was ejected into it. Upon mixing, calcium ions react with carbonates and form calcium carbonates (CaCO_3_), producing visible precipitates within the sessile droplet.

Upon injection of the CaCl_2_ droplet, a distinct vortex-like optical pattern formed within the sessile droplet. This feature is attributed to the refractive-index contrast between the two concentrated salt solutions, together with the impact-induced flow pattern. The vortex-like pattern dispersed and largely disappeared after approximately 30 ms, indicating that this early contrast was associated with the physical mixing process. Following the dissipation of the initial refractive-index pattern, small CaCO_3_ precipitate particles formed were observed to be swirling within the sessile droplet, driven by the residual internal circulation generated by the LiDIA droplet impact. The precipitate particles then gradually increased in size over time, confirming the continued progression of the precipitation reaction after the initial mixing event.

This validates the platform’s utility for precisely timed, volume-controlled reaction initiation on surfaces. The defined impact time provides a clear reaction initiation time point, while the targeted deposition shows that the reagent can be delivered with high spatial control. This capability is directly relevant to drop-on-tape setups in serial crystallography workflows.

**Figure S1.** Drop-on-drop reaction initiation of CaCO_3_ formation: (A) Illustration of the experiment set-up; (B) Key frames from high-speed video recording of the reaction with precipitates highlighted in green

**S.4 Device construction and superhydrophobic coating**

**Figure S2.** Illustration of the construction and superhydrophobic coating process of LiDIA

**Reference**

1.	Waasdorp, R.; van den Heuvel, O.; Versluis, F.; Hajee, B.; Ghatkesar, M. K., *Rsc Adv* **2018,** *8* (27), 14765-14774. DOI 10.1039/C8RA00756J.

2.	Vonderach, T.; Günther, D., Journal of Analytical Atomic Spectrometry 2021, 36 (12), 2617-2630. DOI 10.1039/D1JA00243K.

3.	Harris, D.; Mutz, M.; Sonntag, M.; Stearns, R.; Shieh, J.; Pickett, S.; Ellson, R.; Olechno, J., *SLAS Technology* **2008,** *13* (2), 97-102. DOI <https://doi.org/10.1016/j.jala.2007.12.002>.

4.	DISPENDIX, I.DOT Noncontact Liquid Handling, product brochure, CELLINK/DISPENDIX, 2021, available online: [https://www.cytena.com/products/liquid-handlers/i-dot/](https://www.cytena.com/products/liquid-handlers/i-dot/?utm_source=chatgpt.com) (accessed: May 26, 2026).

5.	FORMULATRIX, MANTIS® Microfluidic Liquid Dispenser, available online: https://formulatrix.com/liquid-handling-systems/mantis-liquid-dispenser/ (accessed: May 26, 2026)

6.	Ansari, A.; Alvarez, R. C.; Karpos, K.; Manatou, D.; Nelson, G.; Nazari, R.; Hochberg, T.; Tamayo, J.; Nockideneh, H.; Hudson, C.; Singhal, A.; Tandon, D.; Forrand, N.; Hu, H.; Doppler, D.; Sonker, M.; Ros, A.; Herrmann, M.; Kirian, R. A., *Lab Chip* **2025**. DOI 10.1039/D5LC00063G.

7.	Lighthill, S. J., *Journal of Sound and Vibration* **1978,** *61* (3), 391-418. DOI https://doi.org/10.1016/0022-460X(78)90388-7.

8.	Bruus, H., *Lab Chip* **2011,** *11* (22), 3742-3751. DOI 10.1039/C1LC20658C.
